# Sustainability is possible despite greed - Exploring the nexus between profitability and sustainability in common pool resource systems

**DOI:** 10.1038/s41598-017-02151-y

**Published:** 2017-05-23

**Authors:** Friedrich Burkhard von der Osten, Michael Kirley, Tim Miller

**Affiliations:** The University of Melbourne, Parkville, VIC 3010 Australia

## Abstract

The sustainable use of common pool resources has become a significant global challenge. It is now widely accepted that specific mechanisms such as community-based management strategies, institutional responses such as resource privatization, information availability and emergent social norms can be used to constrain individual ‘harvesting’ to socially optimal levels. However, there is a paucity of research focused specifically on aligning profitability and sustainability goals. In this paper, an integrated mathematical model of a common pool resource game is developed to explore the nexus between the underlying costs and benefits of harvesting decisions and the sustainable level of a shared, dynamic resource. We derive optimal harvesting efforts analytically and then use numerical simulations to show that individuals in a group can learn to make harvesting decisions that lead to the globally optimal levels. Individual agents make their decision based on signals received and a trade-off between economic and ecological sustainability. When the balance is weighted towards profitability, acceptable economic and social outcomes emerge. However, if individual agents are solely driven by profit, the shared resource is depleted in the long run - sustainability is possible despite some greed, but too much will lead to over-exploitation.

## Introduction

The sustainable use of environmental, social and technical resources has become a significant global challenge^1, 2^. Resource misuse, such as over-fishing^3–5^ or deforestation^6–8^ can potentially result in supply problems and lead to both economic and ecological damage. When the harvesting (or use of) a shared social-economic resource diminishes the value of the resource for other users (negative externality), and it is difficult to control access to the resource in the absence of well-defined property rights (non-excludability), the resource is typically referred to as a *common pool resource* (CPR)^9–12^.

CPR systems are characterized by a social dilemma – the *tragedy of the commons* ^13–15^. That is, the goal of an independently-acting individual is to maximize their use of the resource (gain higher portions of the harvest). However, if all individuals restrained their use of the resource, contrary to their selfish motivations, it should be possible to maintain the resource at a sustainable level, benefiting the population as a whole. An individual’s selfish motivations to reap bigger profits manifest in the implicit assumption that investing more effort into harvesting will gain a larger proportion of the harvest and thus a higher profit, however, this *proportional gains* assumption is never expressed explicitly^16, 17^.

There is a large body of literature describing the management and governance of CPR systems. Perhaps most famous is the pioneering work of Elinor Ostrom^9, 15, 18^, who identified the benefits of managing the commons de-centrally and documented design principles for stable resource management. This work led to substantial related research in the field^19–22^, in laboratory settings^23–26^, as well as via simulation experiments^17, 27–30^. Consequently, a number of external factors have been signalled as acting as drivers for cooperation in the commons, including: communication between individuals^21, 31–33^; punishment of defectors^26, 34–37^; reward^38–40^; trust^14, 41, 42^; social norms^22, 35, 43^; and explicit consideration of the future^25, 44, 45^.

The inherent uncertainties and dynamics of social-ecological systems^26, 46, 47^ exacerbate the social dilemma, as decision-making encapsulates a complex balance of external influences and internal beliefs^17, 19^. Axelrod and others^48–52^ note that cooperation in social dilemmas often comes about because restraint serves both collective and individual interests. When the long-term viability or wealth generation capabilities of individuals (economic entities) is also considered, the issue of ecological sustainability in the commons is more complex^53–57^. Thus, an important question is *“How do individuals realize that restraint is good for their personal interest and the collective interest”*?

In this paper, we depart from the mainstream literature on CPR systems by exploring the nexus between profitability and sustainability when individuals make a harvesting decision in a dynamic common pool resource game. Most models focus on how to prevent selfish individuals from depleting a shared resource, thereby abstracting from the fact that any restraint from resource exploitation may also provide sufficient profit for the population. Typically, the strive for profitability is seen as the underlying problem rather than being seen as a key component of a robust solution to this complex social-ecological dilemma. We remove the implicit proportional gains assumption and replace it with the explicit consideration of profit as a measure for decision-making.

We introduce an integrated mathematical model of a CPR game based on the work of Sethi and Somanathan^30^. Here, individual harvesting decisions are guided by an egoistic component (wealth generation) and an altruistic component (sustainability of the shared resource) dictated by social norms. Akin to the notion of a ‘triple bottom line’^58^, the goal is to integrate both economical and environmental aspects to reach sustainability. Our CPR game is a stochastic model where there are uncertainties associated with the return on harvesting efforts and long-term growth rate of the resource. Individuals can only observe the state of the shared resource and the overall investment of all individuals in the population. We use analytical techniques to derive maximum yields in terms of both sustainability and economics for given game settings. We show that it is possible to derive an analytical solution for optimal harvesting on a system-wide scale, but individual behaviour is difficult to predict, as it depends on the interaction of individuals with each other and the resource.

Given these difficulties, we use numerical simulation experiments to investigate individuals’ harvesting decisions over the long-term. In each round of the game, individuals receive weighted reward signals containing both economical and ecological components. Emergent behaviours are subsequently analysed. We show that individuals can learn harvesting actions that maintain the shared resource at a sustainable level. Importantly, we show that the population as a whole can learn the approximate optimal global behaviour, even when individuals mostly weight their personal profitability over long-run resource sustainability. Individuals learn that long-term sustainability is good for their long-term profit too. We also explore the effects of varying harvesting cost/benefit ratios, finding for instance that when profit margins are too high agents become too greedy and over-exploit the shared resource, thereby providing suggestions on when exogenous intervention might be important to induce an appropriate balance between profit and sustainability motives.

## Model and Analysis

In this section, we introduce our integrated model of the CPR game based on coupled differential equations that capture resource dynamics and individual agent behaviours. The model is presented in stages. First, pure resource dynamics are introduced – the interplay between the growth rate and harvest level of a resource are defined. In the second stage, the sustainability properties of the resource dynamics are determined. In the third stage, the relationship between the ‘investment effort’ agents make to harvesting and the cost and profit are described. In the final stage, all components are integrated into one model and effects of cost and harvesting efforts are explained. An analysis of profitable and sustainable harvesting efforts highlights limitations of this approach and the requirements of the model, which are then translated into goals for individual agents.

### The common pool resource game

In the CPR literature, there are two types of model in which agents simultaneously harvest a resource. The first model focusses on situations where the users of the resource diminish the relative value per resource unit in the current time period as their harvest level increases, however, the future value of the resource is undiminished^59^. In contrast, in the dynamic CPR model the current users of the resource reduce the level of the resource and thereby harm future users of the resource^60, 61^. Uncertainty of resource levels tends to promote over-harvesting^62^, while resource scarcity induces greed^63^. Thus, by explicitly considering resource dynamics on the one hand and macro-economic and social dynamics on the other, a more complete picture can be established.

**Figure 1:**
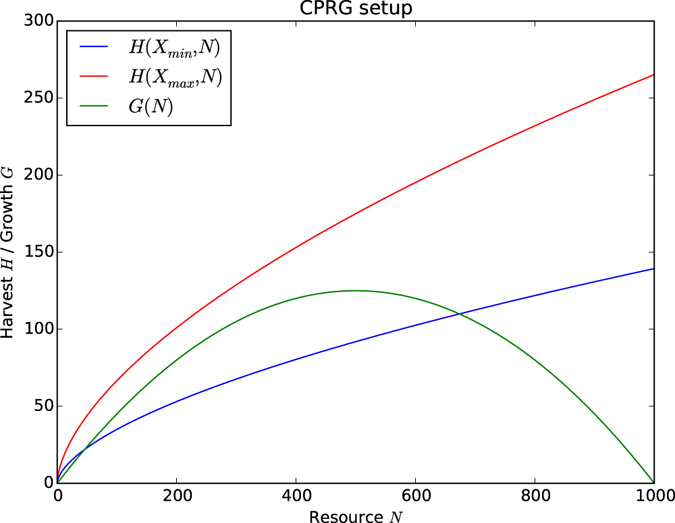
Basic resource dynamics: *G* describes the growth added to the resource depending on the current resource level *N*; *H* describes the harvest taken from the resource, depending on resource level *N* and cumulative effort *X*. Here the limitations of the harvest are shown with the minimum and maximum cumulative effort respectively (*α* = 0.4, *β* = 0.35, *r*_*g*_ = 0.5, *N*_*max*_ = 1,000, see Table 1).

In our CPR game, a group of *n* agents harvest a resource that is expressed as a resource level *N*. The resource has a certain capacity *N*_*max*_ and the resource level may change from time *t* − 1 to *t*:

1 $${N}_{t}={N}_{t-1}+G({N}_{t-1})-H({X}_{t},{N}_{t-1})$$

where *N*_*t*_ is the resource level at time *t*, *G* is the growth level of the resource and *H* is the harvest taken away from the resource. The temporal parameter *t* can represent time, for example days, but we use it purely as an abstraction of time in terms of *rounds* the game is played for. Essentially, the resource changes by its net growth ${\rm{\Delta }}N=G(N)-H(X,N)$ every round. The natural growth *G* that increases the resource stock can take various forms, for example logistic growth (growth level *G* as a function of *N*):

2 $$G(N)={r}_{g}N(1-\frac{N}{{N}_{max}})$$

where *r*_*g*_ is the intrinsic replenishment rate and *N*_*max*_ is the maximum carrying capacity of the resource. Figure 1 shows the growth *G* as a function of the resource level *N* in green.

The harvest function *H*, is modelled using the *Cobb*-*Douglas production function* ^64^:

3 $$H(X,N)=\beta {X}^{\alpha }{N}^{1-\alpha }$$

with constants $\alpha ,\beta \in [0,1]$, where *α* indicates the influence the resource level and invested effort have on the harvesting outcome. The harvest function depends on the cumulated effort *X* invested in the harvest and the state of the resource *N*. The Cobb-Douglas production function is a popular tool in economics to relate capital, labour and the possible outcomes when using the two inputs to produce goods. When modelled onto the CPRG, capital is expressed by the resource *N* and labour is expressed by the invested effort *X*; together they produce the harvest *H*. The function can never produce a negative outcome. In this interpretation the harvest function is a ‘black box’ to agents, that is, an agent cannot predict its exact harvest. A lower *α* value means that the harvest depends more on the resource than on the effort invested, and vice versa for a higher *α*.

The cumulated effort *X* refers to the total effort invested in harvesting the resource. It is a cumulation of the effort *x*_*i*_ of *n* individual agents *i* participating in the game:

4 $$X=\sum _{i}^{n}{x}_{i}$$

In the CPR game, *X* and *x*_*i*_ are limited by upper and lower bounds respectively. Figure 1 shows the minimum and maximum harvest *H* as a function of effort *X* and resource level *N* in blue and red respectively, depending on the upper and lower bounds of *X*.

Each agent *i* can choose an effort *x*_*i*_ to invest. Investing effort is associated with a cost *c* and each agent gets rewarded a pay-off *π*_*i*_ proportional to its investment compared to the combined invested effort *X* of the group:

5 $${\pi }_{i}=\frac{{x}_{i}}{X}H(X,N)-c{x}_{i}$$

where *c* is the constant cost per unit of effort invested and *H*(*X*, *N*) is the harvest (the total payoff in the game is ${\rm{\Pi }}=H(X,N)-cX$). Each agents keeps track of its assets *A*_*i*_ and adds/subtracts its pay-off *π*_*i*_ each round it plays the game:

6 $${A}_{{i}_{t}}={A}_{{i}_{t-1}}+{\pi }_{{i}_{t-1}}$$

where *A* and *π* are an abstraction of real monetary value.

An agent can only observe the state of the resource *N*_*t*_, its invested effort *x*_*i*_, the cumulated invested effort *X*, as well as its pay-off *π*_*i*_ and cumulated pay-off Π, but not the actions of other individual agents nor their pay-offs. An agent can choose how much effort *x*_*i*_ to invest once per round. We later describe in detail how individuals can balance the trade-off between profitability and sustainability goals by explicitly weighting objectives (denoted by *λ* when considering sustainability, and *ξ* when considering personal assets) when making a harvesting decision. Table 1 details the CPRG parameters used in the simulations.

**Table 1:** CPRG parameters used in the experiments.

Parameter	Value
*N*_*max*_	1000
*r*_*g*_	0.5
*α*	0.35
*β*	0.4
*X*_*min*_	100
*X*_*max*_	500
*c*	0.5

### Analysis

An agent participating in the CPR game must balance two possibly conflicting goals across multiple rounds of the game: (a) help to maintain the resource at a sustainable level, which is an inherent global goal; and (b) maximize its own profit (accumulate assets).

#### Sustainability

A common objective of CPR systems is to maintain a ‘steady level’ of the resource, thereby allowing ‘large’ yields over an indefinite period under constant environmental conditions. In our model, the global sustainability goal, *λ*, entails harvesting the resource over time in a manner such that the resource growth level *G* is at least equal to the harvest *H* or higher, so that the resource stock level *N* does not decline and the resource *net growth G* − *H* is non-negative. The population as a whole can regulate the harvest via the effort *X* invested. The boundary as to what level of effort yields a sustainable harvest is given by the Maximum Sustainable Yield (MSY):

7 $$\begin{array}{llll}MSY(N) & = & \mathop{{\rm{\max }}}\limits_{X\in [{X}_{min},{X}_{max}]} & G(N)-H(X,N)\\  & = & \mathop{{\rm{\max }}}\limits_{X\in [{X}_{min},{X}_{max}]} & {r}_{g}N(1-\frac{N}{{N}_{max}})-\beta {X}^{\alpha }{N}^{1-\alpha }\end{array}$$

**Figure 2:**
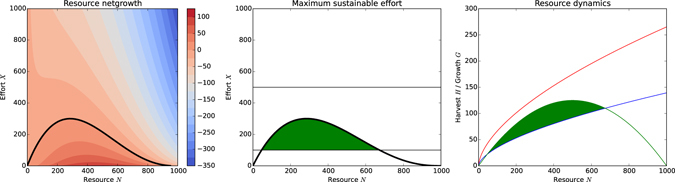
Suiatainable harvest levels. (**a**) Contour plot of resource net growth *G* − *H*; the black line indicates ${X}_{{\max }}^{{sustainable}}$, efforts above this line produce negative net growth. (**b**) Resource net growth with effort limits (thin black lines); the green shaded area emphasises sustainable effort levels at which the net growth *G* − *H* ≥ 0. (**c**) Resource dynamics with positive net growth resulting from the sustainable harvest efforts described in Fig. 2b indicated by the green shaded area.

For the resource *net growth G* − *H* to be positive, we can derive the condition

8 $${X}_{{\max }}^{{sustainable}}\le {(\frac{{r}_{g}N(1-\frac{N}{{N}_{max}})}{\beta {N}^{1-\alpha }})}^{\frac{1}{\alpha }}$$

which is an upper bound on the cumulative effort invested to prevent resource stock levels from declining. Figure 2a shows the resource net-growth with Fig. 2b detailing the sustainable effort levels bounded by the MSY and the minimum and maximum effort that an agent can invest. Figure 2c illustrates the MSY integrated into the resource dynamics; the green shaded area indicates sustainable harvests based on the effort levels in Fig. 2b. An individual agent *i* can influence the cumulative effort *X* via contribution of *x*_*i*_ but there is no clear definition of a boundary on *x*_*i*_ for the MSY, since the cumulative effort *X* depends on the other agents in the population and we assume that agents act independently. In addition, the impact of individual actions diffuses with higher numbers of individuals participating in the game. This action diffusion and the lack of a defined boundary for *x*_*i*_ make the goal of sustainability harder to control for an individual agent. It is clear however, that a lower investment of effort results in less harvest being extracted and thus higher *net growth* of the resource.

#### Profitability

A dominant economic standpoint is to simply view the shared resources as a type of asset, which should be managed so as to maximize its value to society. In formal terms, an agent *i* wishing to maximize its profit *π*_*i*_ to fulfil its personal goal *ξ* has to maximize the difference between investment (effort) and return (harvest); see Equation (5). The effort leading to the highest profitability for the entire CPR game system can be described by the Maximum Economic Yield (MEY):

9 $$\begin{array}{llll}MEY(N) & = & \mathop{{\rm{\max }}}\limits_{X\in [{X}_{min},{X}_{max}]} & H(X,N)-cX\\  & = & \mathop{{\rm{\max }}}\limits_{X\in [{X}_{min},{X}_{max}]} & \beta {X}^{\alpha }{N}^{1-\alpha }-cX\end{array}$$

Note that we are optimizing for total profit, not profit per unit of effort (which would require division by *X*). For the profit to be positive, i.e. *H* − *cX* ≥ 0, we can derive the condition

10 $${X}_{{\max }}^{{profitable}}\le {(\frac{\beta {N}^{1-\alpha }}{c})}^{\frac{1}{1-\alpha }}$$

**Figure 3:**
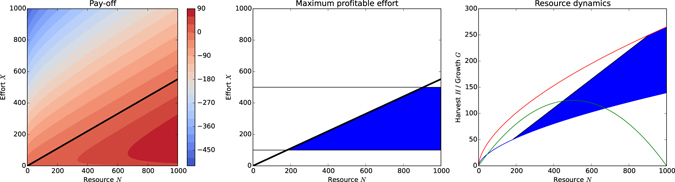
Profitable harvest (when cost *c* = 0.5). (**a**) Contour plot of cumulative profit *H*(*X*, *N*) − *cX*; the black line indicates ${X}_{{\max }}^{{profitable}}$, efforts above this line produce negative profit. (**b**) Profit with effort limits (thin black lines); the blue shaded area emphasises profitable effort levels at which the total profit *H*(*X*, *N*) − *cX* ≥ 0. (**c**) Resource dynamics with positive profits resulting from the profitable harvest efforts described in Fig. 3b indicated by the blue shaded area.

An individual agent however, will want to maximize its individual profit instead of the cumulative profit of the population:

11 $$\mathop{{\rm{\max }}}\limits_{{x}_{i}\in {\mathscr{A}}}\,\frac{{x}_{i}}{X}\beta {X}^{\alpha }{N}^{1-\alpha }-c{x}_{i}$$

where ${\mathscr{A}}$ is the set of actions available to an agent. Figure 3a shows the profit yield levels across resource and effort levels with Fig. 3b identifying positive profits that are bounded by the MEY and minimum and maximum effort. Figure 3c again shows the MEY integrated into the resource dynamics, where the blue shaded area indicates profitable harvests based on the effort levels in Fig. 3b.

#### Goals

**Figure 4:**
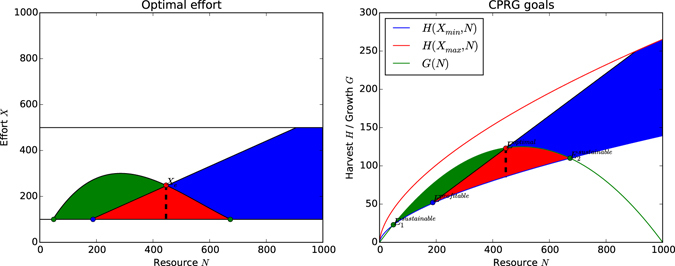
Integrating Figs 2 and 3 into a full socio-ecological system. The green and blue dots are the sustainability and profitability equilibria respectively, i.e. the resource levels below or above which sustainable/profitable harvest is not possible any more. The red dot is the optimal equilibrium, at which the largest volume of distinct actions leads to sustainable and profitable harvest. (**a**) The bounds for effort levels that yield a profitable AND sustainable harvest are denoted by the red shaded area. (**b**) Resource dynamics with profitable and sustainable harvesting indicated by the red shaded area.

What is the optimal effort an agent can invest in order to fulfil both its individual goal, *ξ*, and the collective goal, *λ*? The sustainable and profitable effort levels bounded by MSY and MEY respectively are shown in Fig. 4a and intersect in the red shaded area. Figure 4b shows sustainable and profitable harvest levels with respect to the level of the resource, as determined by the effort levels in Fig. 4a. Note that the red shaded area only makes for 7% of the possible choices for investing effort. The parameters of the game have been chosen such that only this relatively small pool of actions is viable, otherwise it would be too easy for agents to harvest sustainably and profitably, as many actions lead to that outcome. Alternative setups for the CPRG can be found in ref. 65 where almost any action is sustainable and profitable. Figure 4b also shows ${E}_{1}^{{sustainable}}$ and ${E}_{2}^{{sustainable}}$ the minimum and maximum equilibria at which the harvest is equal to the growth. Equivalently, *E* ^*profitable*^ is the minimum equilibrium at which harvest becomes profitable, i.e. is at least equal to the invested effort.

Let *X*_*e*_ denote the optimal *X* that produces sustainable and profitable harvests in the red shaded area. The optimal equilibrium *E*_*optimal*_ describes the resource level at which most actions *X*_*e*_ produce viable harvests, it is thus the ‘safest’ equilibrium due to robustness against individual agents taking unsustainable or unprofitable actions. Ideally, each agent takes an action around $\frac{{X}_{e}}{n}$ such that the harvest from cumulative effort lies close to *E*_*optimal*_. This action is then denoted by *x*_*e*_.

An agent *i* now has to find an action *x*_*i*_ that contributes to *X* in such a way that *X* satisfies the condition in Equations (8) and (10) and is as close to *X*_*e*_ as possible. This presents the *tragedy of commons* ^13^: each individual agent would fare better if all agents restrained their use but the interest of an individual agent is to maximize use in order to gain higher portions of the harvest given the behaviour of other agents does not change, i.e. maximize $\frac{{x}_{i}}{X}$ without changing *H* as seen in Equation (11). Ideally, ${x}_{e}=\frac{{X}_{e}}{n}$ but *x*_*e*_ cannot be derived analytically, since actions of other agents cannot be accounted for.

Note that there is a minimum investment ${X}_{min} > 0$ in our game model (see Table 1). The stochastic CPRG is different from the Public Goods Game or other social dilemmas such as the Prisoner’s Dilemma, in that participation is not voluntary^66^. An investment of *x*_*i*_ = 0 essentially means that agent *i* is not participating in the game. Furthermore, in other social dilemmas cooperation prevents collective loss only when players contribute significantly^67^. Significant contributions in the CPRG almost certainly lead to over-harvesting of the resource. Consider a scenario where an agent’s asset declined each round (possibly corresponding to a ‘living expense’). If they do not invest *x*_*i*_ > 0, thus taking a risk of investing effort in harvesting the resource, the inevitable conclusion is that they do not survive economically. However, individual self-interest is not always against cooperation, as it is in the Prisoner’s Dilemma. Typically, studies of social dilemmas assume that individuals playing the game have a discrete choice, either to ‘cooperate’ or to ‘defect’. Of course, the tragedy of commons can be expressed from a game theoretic perspective as well^68^. In that case, cooperation and defection are clearly defined as binary actions. However, this binary decision is somewhat unrealistic, especially when investment levels are considered in games such as the CPRG. We suggest that individual game playing agents are diverse and subsequently can make different decisions when confronted with variance in outcomes. Therefore, we extend the actions available to an individual by taking into account the range of the strategy space^69^.

#### Cost

**Figure 5:**
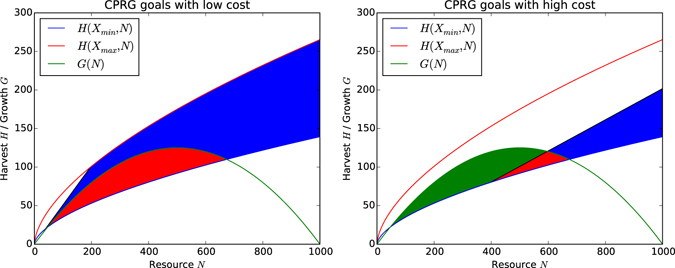
The influence of cost on resource dynamics and the ability to harvest profitably and sustainably. (**a**) A CPRG setup with cost *c* = 0.2; almost any action becomes profitable, but only a subset of actions is sustainable. Agents will not be able to distinguish between the two anymore and start overharvesting. (**b**) A CPRG setup with cost *c* = 0.8; the set of actions that are both profitable and sustainable becomes very small. Agents will not be able to make profits as they need to maintain the resource at a high level.

In addition to the effort agents invest in harvesting, the cost incurred on that investment is a major factor that influences how individuals will act in the game. Figure 5a demonstrates the change in sustainability and profitability requirements when the constant cost per effort is lowered. Almost any action now results in a profitable harvest (blue shaded area), including all sustainable actions (red shaded area). This makes it hard for agents to distinguish between them when considering profit for their decision making, even when taking into account sustainability to some extent. This scenario will more than likely result in depletion of the resource. On the other hand, Fig. 5b demonstrates the change in sustainability and profitability requirements when the constant cost per effort is increased. Only a few actions result in both sustainable and profitable harvest, making it difficult for agents to select profitable actions. This scenario will more than likely result in a sustained resource but asset losses for the agents. Hence, the cost is a major factor when regulating a CPR and needs to be balanced in order to allow for sustainable harvesting in the first place.

### Simulation experiments

This section describes the numerical simulation experiments conducted to test the hypothesis that individuals can learn to harvest a resource sustainably by considering profit when making a harvesting decision. First, an individual’s harvest decision-making is explained, then the results of the simulations are shown.

### Harvest decision-making

There are many different mechanisms that an individual might adopt when faced with a harvesting decision. Assuming ‘rational resource users’, expected utility theory could be used to guide the decision-making process. Evolutionary game theory and imitation (social) learning, widely used techniques employed when tackling social dilemmas such as the public goods game, are not appropriate in our stochastic implementation of the CPR game as they tend to converge very slowly^70^.

Even though the dynamics of the CPRG are deterministic, we look at it as a Markov decision process from an individual agent’s point of view. Given the definition of the CPR game, a Markov decision process is a suitable representation of the game. Reinforcement learning is a well-known learning mechanism tailored for Markov decision processes and has the added benefit of being a decentralized individual learning method^71, 72^. Since the goal of this paper is to influence individual behaviour, Q-Learning^73^, an implementation of Reinforcement Learning, has been selected to guide decision-making. Based on the two goals of an agent defined in the analytic Section, agents in the simulation have two motivations that influence their choice of actions. Social norms are rules of behaviour that are considered acceptable in a society and emerge from that society itself^74^. Thus, we assume that sustainability is a norm that society has developed to consider for different reasons. An agent might want to conform to a social norm to be responsible, or perhaps because they care for the environment, or they realize that restraint helps preservation of the population. *λ* represents the collective sustainability goal motivated by social norms

12 $$\lambda =\{\begin{array}{lll}1 & {\rm{if}} & {{\rm{\Delta }}}_{N} > 0\\ 0 & {\rm{if}} & {{\rm{\Delta }}}_{N}=0\\ -1 & {\rm{if}} & {{\rm{\Delta }}}_{N} < 0\end{array}$$

As for the wealth generation goal, it is based on a model proposed by Van Lange^75^ who argues that people’s interests include different social values including selfishness and equality. He proposes an integrative model of social values that transforms those values into a single utility value. Whereas traditional economic models focus on self-interested behavior and social value orientations research focuses on the population differences, Van Lange postulates that people’s behavior is best understood as maximizing pro-self and pro-social behaviors, only in different combinations (this is applied in Equation (14)). Furthermore, pro-social behavior is not simply the altruistic interest in maximizing others’ outcomes, but also includes interest in the equality of outcomes. The equality of outcomes in this case is the pay-off of the population as a whole. *ξ* corresponds to the individual goal of wealth generation (the profit of the individual agent and the population as a whole)

13 $$\xi =\{\begin{array}{lll}1 & {\rm{if}} & {\pi }_{i} > 0\\ 0 & {\rm{if}} & {\pi }_{i}=0\\ -1 & {\rm{if}} & {\pi }_{i} < 0\end{array}$$

Q-Learning allows an agent to learn from feedback from their environment. In our extension of Q-Learning, an individual agent incorporates the feedback from its own actions, and from the accumulated actions of all participants of the game. The actions an agent takes result in a weighted reward *R* that measures both the profitability and sustainability of the action. We define the reward as:

14 $$R=w\xi +(1-w)\lambda $$

where the weight $w\in [0,1]$ determines to what extent an agent considers one or the other component. The reward *R* presents an unbiased evaluation of the environmental state of an agent as a response to its actions, incorporating consideration for both collective and individual goals according to Van Lange’s model of integrative social value. Note that both components *ξ* and *λ* are given as trends only (−1 for decline, 0 for no change, 1 for increase). Since the magnitudes of the two measures vary considerably (*λ* ∈ [−1,000, 1,000, *ξ* depends on the number of agents in the game, for example *ξ* ≈ ∈[−5, 5] for 10 agents), they would impact the reward differently.

The population stays the same throughout the simulations. Within game theory there are two population dynamics metaphors. The biological one assumes that individuals die out and get replaced over time (e.g. evolutionary algorithms)^76^, while the economic one assumes that individuals change their strategy over time (e.g. social learning)^77^. As agents are assumed to act independently in our simulations, and the biological basis of population dynamics are not relevant, the simplified economic perspective was adapted. The change of strategies takes place via the Q-Learning mechanism that changes the action selection probability distribution over time.

### Simulation parameters

All experiments have been run with the same setup of the CPR game described in Fig. 1. Each experiment was run for 5,000 rounds and repeated 50 times to generate reliable statistical data. Agents are endowed with assets *A* = 1,000 initially. In the first experiment, the number of agents was increased ∈[1, 15], to demonstrate the agents’ ability to fulfil both goals while harvesting. Experiments with larger groups have been conducted, but the self-efficacy problem prevents solutions from being effective (see results), which should be addressed in separate research. In the second experiment, the cost *c* ∈ [0.1, 09] and reward weight *w* ∈ [0.1, 09] are varied in steps of 0.1 to demonstrate the influence of the cost on the ability of agents to successfully fulfil both goals as predicted in the previous Section, and to examine to what extent an agent can consider profit to fulfil its goals. Note that a weight of *w* = 0 represents the assumption that only the component *ξ* (the individual motivation for profit) guides agent behaviour, whereas a weight of *w* = 1 represents the assumption that only the component *λ* (the global motivation for sustainability) guides agent behaviour. In the case of *ξ* this would mean agents are purely profit oriented and only act to increase their profit regardless of the state of the resource. In the case of *λ* this would mean that agents only consider sustainability regardless of their own profits or losses. Van Lange has also suggested that the integrative model mentioned in the previous section provides a parsimonious explanation for individual decisions that are only rational at the level of the group^78^. It follows that despite extreme cases, individuals will mostly consider both components to some extent. Table 1 details the CPRG parameters used in the simulations.

## Results

**Figure 6:**
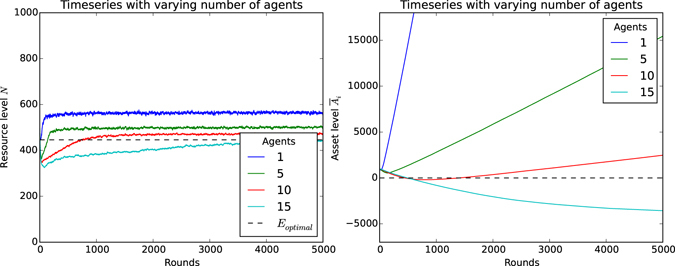
Time-series plots with varying number of agents and weight *w* = 0.5. Error bars have been omitted as the values are very small. The CPR game parameters are listed in Fig. 1. (**a**) The development of the resource level *N* over time with increasing number of agents. The black dotted line denotes the equilibrium *E*_*optimal*_ derived in the analysis. (**b**) The development of the asset level *A* per agent with increasing number of agents. The black dotted line denotes the threshold for losses, i.e. *A* < 0.

Figure 6a plots time series values for the resource level over time, when different sized groups are harvesting the resource. Figure 6b details the corresponding development of the average assets of individual agents. Note that in these experiments, equal weights were assigned to both components of the reward function, i.e. agents consider both their individual goal by means of profit and the collective goal by means of resource level to equal parts.

**Figure 7:**
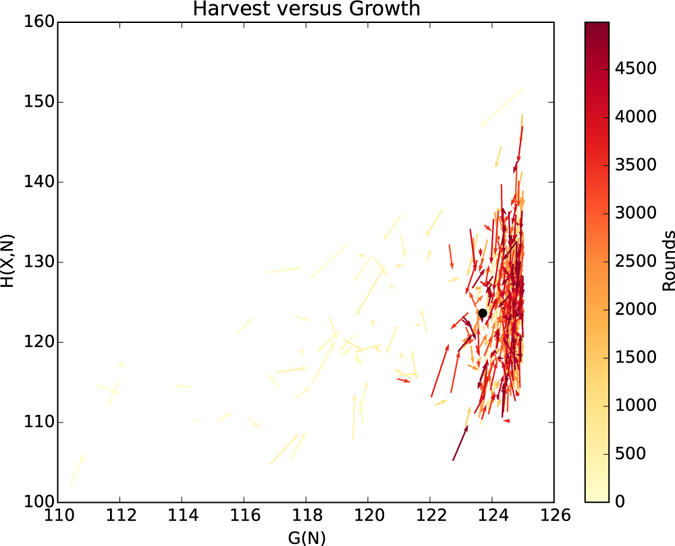
A phase graph plotting harvest versus growth demonstrates how the population as a whole harvests in a manner that moves the resource level towards *E*_*optinal*_ (the black dot). After the initial learning phase (brighter arrows), agents settle oscillating around the optimal resource level (dark arrows), indicating they invest efforts described in Fig. 4.

The plot in Fig. 6a shows that after an initial learning phase, agents harvest the resource in a stable and sustainable manner, while their assets increase. Importantly, agents have learnt to make a profit and harvest sustainably, considering profit as a measure of self-interest. The time series plot of the development of assets in Fig. 6b shows that if the population harvesting a resource grows too large, they are no longer able to maintain a profit. This effect is more than likely caused by reduced self-efficacy, as the link between an agent’s action and the resulting environmental response becomes more ambiguous with more participants, i.e. an individual action has less influence on the global harvesting outcome such that they cannot learn the intended behaviour any more. Solving the self-efficacy problem is an interesting approach for future research. Not only do agents learn to harvest sustainably and profitably, the resource levels are close to the global optimum derived in the analysis and shown in Fig. 4. This is further corroborated in Fig. 7.

The harvests in Fig. 7 tend to follow the growth of the resource, indicating that agents adapt their harvesting behaviour towards the optimal resource level, as they learn that it gives them the highest likelihood of a profit. The plot reveals that the behaviour of the population as a whole has a tendency to oscillate within the bounds of sustainable and profitable harvest (around the optimal equilibrium denoted by the black dot), as indicated by the red shaded area in Fig. 4b. The question arising now is whether an agent must consider both profit and resource level equally when making a harvesting decision.

**Figure 8:**
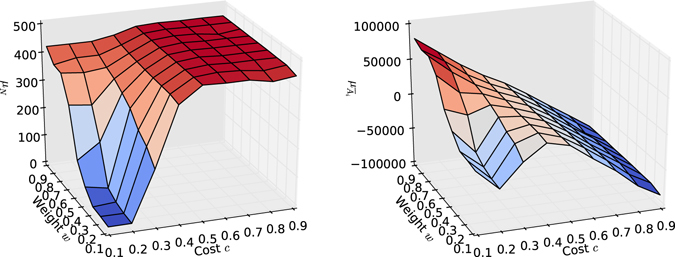
Varying cost *c* and weight *w* with a population of 10 agents after 5000 rounds. The CPR game parameters are listed in Fig. 1. (**a**) The influence of cost and weight on the resource level *N*; low costs induce greed, whereas low weights can be surprisingly sustainable. (**b**) The influence of cost and weight on the asset level *A*; profits decrease with cost, but over-harvesting also diminishes profits due to resource depletion.

In the plots shown in Fig. 8, both the cost of investing effort and the weight balancing consideration of profit and resource have been varied. The results confirm the predictions made in the analysis and shown in Fig. 5; a low cost leads to more greed, resulting in a depleted resource, whereas high costs make it almost impossible to yield a profit, resulting in declining assets. The cost influences the risk for an individual participating in the game. The ‘risk orientation’ of an individual may be seen as an individual’s general preference towards making decisions in uncertain situations. From a social psychological science perspective, people tend to be risk-averse when dealing with outcomes that are gains relative to their reference point—they choose sure smaller gains over larger riskier gains—but become risk seeking when dealing with losses^79^. This is in contrast to the widely accepted economic perspective, where a rational decision-maker is an individual who attempts to maximise their expected utility in any decision-making scenario. In the case of Reinforcement Learning applied to the CPRG, the risk decreases with a lower cost and higher investments become less risky, as the expected reward becomes positive even for high investments. Thus the profit component of the Reinforcement Learning reward signal *R* is more dominant and steers the behaviour of agents towards greediness.

Figure 8 indicates that agents can discount their global goal of sustainability to a rather surprising extent, unless the cost parameter makes it difficult to distinguish profitable and sustainable harvesting efforts. The reason for this seems to lie in the fact that considering profit explicitly actually teaches an agent that sustainability has a significant influence on how profitable harvests are in the long run. If agents solely consider their profits for harvesting decisions they fail to harvest sustainably nor profitably, as no distinction between sustainability and profitability takes place. This result indicates that sustainability is related to cost. When the cost is too high, almost all actions an agent takes lead to a loss. Thus the sustainability component of the Reinforcement Learning reward signal *R* becomes the dominant factor, steering agents towards sustainability. Only in situations with a reasonably low cost allowing profits to be made, sustainability is proportional to the weight placed on it. In the overall picture, the cost influences to what extend agents can discount their sustainability goal, i.e. the higher the cost, the less emphasis needs to be placed on sustainability and vice versa. In light of the cost inducing greed, this makes sense, as the emphasis on sustainability counteracts greed.

## Conclusion

In this paper, we have examined the dynamics of an integrated mathematical model of a CPR game incorporating both economic and ecological sustainability criteria. Analytical analysis was used to derive values for the maximum sustainable and economic yields, as well as the optimal harvest effort. Numerical simulation experiments were then used to investigate the efficacy of a reinforcement learning algorithm used to determine the effort each agent allocated to harvesting. Agents learn what actions to take by explicitly considering profit and resource sustainability as objectives instead of relying on the proportional gains assumption. They are able to make complex harvesting decisions independently of environmental or inter-individual influences. Despite the complexity of the agents’ decision making, the model is simple enough to isolate the factors that lead individuals to cooperate.

Our results confirm that when profit gained is used as part of the harvesting decision-making process both resource sustainably and economic survival of the participating individuals is possible. The results also place further importance on the fact that the cost of harvesting a resource has a significant impact on any kind of harvesting behaviour, which in turn can determine success or failure of harvesting endeavours. Significantly, economic and ecological trade-off characteristics of CPR systems with complex dynamics can be captured relatively easily in our integrated model.

It would be interesting to follow up insights gleaned from this theoretical paper in the field. In particular, experiments could be devised to test whether it is feasible for individuals harvesting a resource to make sustainable decisions even if they are not concerned with sustainability *per se*. In addition, further analysis of the effects of the harvesting group size should be explored.

Returning to the title question: *“Is greed good”*?, we find that sustainable harvesting of resources is possible despite explicit consideration of profit. In particular, it is interesting that individuals need not give up their concerns for profit entirely as long as they have at least some concern for sustainability.
